# Evaluating Short-Term Patient-Reported Outcome Measures Following Total Hip and Knee Replacement: A Comprehensive Review

**DOI:** 10.7759/cureus.70468

**Published:** 2024-09-29

**Authors:** Vatsal Patel, Sanjay V Deshpande, Vivek H Jadawala, Disheeta Bhalsod, Sharad Sawant

**Affiliations:** 1 Orthopedics, Jawaharlal Nehru Medical College, Datta Meghe Institute of Higher Education & Research, Wardha, IND; 2 Anesthesiology, Jawaharlal Nehru Medical College, Datta Meghe Institute of Higher Education & Research, Wardha, IND

**Keywords:** patient-reported outcome measures (proms), postoperative recovery, quality of life, short-term outcomes, total hip replacement (thr), total knee replacement (tkr)

## Abstract

Total hip replacement (THR) and total knee replacement (TKR) are widely performed surgical procedures to alleviate pain and improve function in patients with joint-related diseases. Short-term patient-reported outcome measures (PROMs) have become a key metric in assessing the success of these surgeries from the patient's perspective, focusing on early recovery, pain management, mobility, and quality of life. This comprehensive review evaluates the significance of short-term PROMs following THR and TKR, highlighting commonly used tools such as the Oxford Hip Score (OHS), Oxford Knee Score (OKS), Western Ontario and McMaster Universities Osteoarthritis Index (WOMAC), and short-form health survey (SF-36). The analysis explores the impact of various factors, such as age, preoperative health status, and surgical technique, on short-term outcomes. Findings from recent studies indicate that while patients generally report improvements in physical function and pain relief within the first six months post-surgery, individual outcomes can vary significantly. Factors like early rehabilitation, mental health, and the presence of postoperative complications can influence the trajectory of recovery and satisfaction levels. Moreover, the review addresses the limitations of current PROMs, including variability in reporting and sensitivity to different patient populations. This review emphasizes the need for more personalized and standardized approaches to PROM assessment to better capture patient experiences and optimize postoperative care. Future research should focus on integrating PROMs with long-term follow-up data and digital health tools to track real-time patient progress, thus enhancing the overall quality of care for THR and TKR patients. Short-term PROMs play a vital role in understanding patient outcomes and guiding clinical practice for joint replacement surgeries.

## Introduction and background

Total hip replacement (THR) and total knee replacement (TKR) are among the most common and successful orthopedic surgeries performed worldwide [1]. These procedures aim to alleviate pain, restore function, and improve the quality of life (QoL) for patients suffering from joint-related issues, primarily due to osteoarthritis, rheumatoid arthritis, or trauma. With the aging global population and increasing prevalence of joint diseases, the demand for THR and TKR has grown substantially [2]. The number of joint replacements is projected to rise significantly in the coming decades, driven by aging demographics and advancements in surgical techniques and technologies [3]. Assessing the outcomes of these surgeries is crucial, as patient satisfaction and functional recovery are directly tied to the success of the procedures. In particular, short-term outcomes, such as early pain relief, mobility improvement, and overall recovery, play a key role in determining the effectiveness of the intervention and guiding postoperative care [4]. A comprehensive evaluation of these outcomes is necessary to ensure that the surgeries meet patients' expectations and the healthcare system's quality standards [4].

Patient-reported outcome measures (PROMs) are valuable tools in healthcare that capture the patient's perspective on their health, functional abilities, and quality of life. PROMs allow for the assessment of subjective outcomes, such as pain levels, physical functioning, and emotional well-being, which may not be adequately captured by clinical or objective measures alone [5]. The use of PROMs in healthcare has expanded in recent years, particularly in surgical fields like orthopedics, where patient satisfaction and recovery are central to evaluating success [5]. PROMs play a crucial role in assessing postoperative outcomes in joint replacement surgeries. They provide insights into how patients perceive their recovery, including improvements in pain, mobility, and overall quality of life [6]. Using standardized PROM tools, clinicians can track patient progress, compare outcomes across different populations, and make informed decisions about patient care. Additionally, PROMs contribute to the growing emphasis on patient-centered care, where the patient's experience and voice are integral to the overall assessment of treatment effectiveness [6].

The primary objective of this review is to evaluate the short-term patient-reported outcome measures (PROMs) following total hip replacement (THR) and total knee replacement (TKR). By focusing on the early stages of recovery, this review aims to provide a comprehensive understanding of the factors influencing patient satisfaction, functional improvement, and quality of life in the immediate postoperative period. Furthermore, it will explore the key PROMs used in these surgeries, the outcomes they measure, and the implications for clinical practice. Through a detailed analysis of current evidence, this review will highlight areas for improvement in PROM assessments and suggest future directions for optimizing patient outcomes after joint replacement surgeries.

## Review

Background: THR and TKR

THR and TKR are among the most commonly performed surgical procedures designed to alleviate pain and restore function in patients with advanced osteoarthritis [7]. These surgeries are typically recommended for individuals who suffer from severe joint pain and functional limitations that significantly diminish their quality of life. The primary conditions leading to THR and TKR include end-stage osteoarthritis, rheumatoid arthritis, post-traumatic arthritis, and avascular necrosis of the femoral head. These conditions result in progressive degeneration of the articular cartilage and underlying bone, leading to pain, stiffness, and restricted mobility [8]. Over the years, surgical techniques for THR and TKR have seen substantial advancements. In THR, the damaged hip joint is replaced with a prosthetic implant, typically consisting of a metal or ceramic ball (femoral head) and a metal or plastic socket (acetabulum). Various surgical approaches, such as the posterior, lateral, and anterior techniques, are utilized, each with distinct advantages and drawbacks [9]. Likewise, TKR involves replacing the damaged knee joint with artificial components. Common surgical approaches for TKR include the medial parapatellar, midvastus, and subvastus techniques. Recent innovations, including minimally invasive methods, computer-assisted navigation, and robotic-assisted surgery, have been developed to improve precision, reduce recovery time, and enhance overall surgical outcomes [10]. Short-term outcomes following THR and TKR are critical for assessing the success of these procedures. PROMs provide valuable insights into pain levels, functional mobility, and overall patient satisfaction in the early postoperative phase. Monitoring these short-term outcomes is essential for evaluating recovery progress and identifying factors influencing patient recovery [11]. Additionally, early assessments help guide rehabilitation strategies to optimize recovery and improve patient satisfaction. Focusing on these early indicators allows healthcare providers to make data-driven decisions that enhance the patient experience and outcomes in joint replacement surgeries [11].

Understanding PROMs

PROMs are crucial for assessing the effectiveness of THR and TKR surgeries. PROMs provide valuable insights into patients' perspectives on their health, functional capabilities, and overall satisfaction with surgical outcomes. By capturing the patient's voice, these measures allow healthcare providers to evaluate the impact of surgical interventions on quality of life [12]. Several widely used PROMs are employed in evaluating THR and TKR. The Oxford Hip Score (OHS) is designed to assess hip-related issues, focusing on pain and function. While its strength lies in its targeted approach, it may not fully capture broader aspects of health-related quality of life [13]. Similarly, the Oxford Knee Score (OKS) focuses on knee function and pain, making it a staple in clinical evaluations. However, like the OHS, it may overlook other significant health domains. The Western Ontario and McMaster Universities Osteoarthritis Index (WOMAC) comprehensively assesses pain, stiffness, and physical function in osteoarthritis. Still, its primary focus on this condition may limit its applicability to other patient groups [14]. In contrast, the short-form health survey (SF-36) is a generic tool that assesses a range of health domains, including physical and mental health. Although it provides a broader perspective on overall health status, it may be less sensitive to changes specific to hip or knee conditions than condition-specific PROMs [15]. PROMs assess several key domains essential for understanding patient outcomes post-surgery [16]. These domains include physical function, which measures the ability to perform daily activities; pain relief, which evaluates the intensity and interference of pain in everyday life; quality of life, which examines overall well-being and life satisfaction post-surgery; mental health, which assesses factors like anxiety and depression that may influence recovery; and patient satisfaction, which reflects overall contentment with surgical outcomes. By evaluating these domains, healthcare providers can comprehensively understand patients' recovery experiences [16]. The distinction between short-term and long-term PROMs is important when assessing surgical outcomes. Short-term PROMs, typically evaluated within the first six months after surgery, focus on immediate recovery, such as pain relief and functional improvement. In contrast, long-term PROMs assess outcomes over a year or more, focusing on sustained improvements in quality of life and long-term satisfaction with the surgical intervention [17]. Short-term evaluations are particularly valuable for identifying early complications, assessing pain management strategies, and gauging the initial impact of surgery on functional abilities. This timely feedback allows healthcare providers to implement necessary interventions if patients experience suboptimal recovery [18].

Key short-term PROMs in THR and TKR

Key short-term patient-reported outcome measures (PROMs) following THR and TKR encompass critical aspects of recovery, such as physical function, pain relief, quality of life (QoL), and patient satisfaction. Each area provides valuable insights into the effectiveness of these surgical interventions and the overall patient experience [19]. One of the most significant outcomes reported by patients after THR and TKR is improved mobility and activity levels. Studies consistently show marked enhancements in physical function post-surgery, often evaluated through standardized tests like the six-minute walk and chair stand tests [20]. For instance, TKR patients frequently report substantial gains in walking endurance compared to their preoperative condition. However, certain factors may hinder recovery, including pre-existing comorbidities, higher body mass index (BMI), and age-related declines. Research indicates that over 20% of patients do not experience significant functional improvement after TKR, highlighting the need for individualized rehabilitation programs to meet specific patient needs [21]. Pain relief is another crucial short-term outcome following THR and TKR. Both procedures have demonstrated significant reductions in pain levels postoperatively. Patients often report lower pain scores following surgery than preoperative assessments [22]. For example, studies show a 12% reduction in pain scores following TKR, reflecting substantial improvements in bodily pain. However, a portion of patients continue to experience persistent pain, underscoring that while surgery provides considerable relief, it may not eliminate discomfort for all individuals [23]. THR and TKR also have a profound impact on quality of life. Both surgeries significantly improve patients' psychological well-being and social functioning. Enhanced physical function often leads to better QoL metrics, as patients can engage in daily activities and social interactions after surgery [24]. These improvements, including enhanced abilities to perform tasks like walking, climbing stairs, and participating in recreational activities, are vital for maintaining independence and overall well-being [24]. Finally, patient satisfaction rates following THR and TKR are generally high. Numerous studies reveal that most patients express satisfaction with their surgical outcomes, attributing improved quality of life to effective pain management and restored mobility [25]. However, satisfaction levels are influenced by factors such as preoperative expectations, recovery time, and individual health profiles. Patients with realistic expectations about recovery tend to report higher satisfaction rates, while those who experience faster recoveries often express greater contentment with their overall results [25].

Factors influencing short-term PROMs

Several key factors influence short-term PROMs following THR and TKR surgeries, each playing a crucial role in determining the quality of recovery and patient satisfaction [26]. Patient-specific factors significantly affect recovery outcomes. Age is a major determinant, as older patients often have different recovery trajectories compared to younger individuals due to variations in bone quality, healing capacity, and physical resilience [27]. Gender also plays a role, with research indicating that women may face higher risks of complications and report lower satisfaction levels after surgery. Preoperative health status is another critical factor; patients with comorbidities such as obesity, diabetes, or cardiovascular diseases generally report poorer outcomes. Furthermore, mental health is essential in predicting recovery, as psychological factors like preoperative anxiety and depression have been associated with lower satisfaction and less favorable outcomes post-surgery [27]. Surgical factors also play a pivotal role in shaping short-term PROMs. The type of implant used can influence both the longevity and stability of the prosthesis, which, in turn, affects patient outcomes. For example, the choice between cemented and uncemented implants may result in different recovery experiences and levels of satisfaction [28]. The surgical approach-anterior or posterior-can impact recovery times, pain levels, and overall patient satisfaction. Additionally, the surgeon’s expertise with a particular technique minimizes complications and ensures smoother recoveries. Effective postoperative care, which includes pain management and close monitoring for complications, is equally important in promoting optimal recovery and enhancing patient satisfaction [29]. Rehabilitation and physical therapy are essential for improving PROMs after surgery. Early postoperative rehabilitation is critical for maximizing functional recovery. Tailored rehabilitation programs that account for individual patient characteristics significantly enhance outcomes. Studies show that structured physical therapy interventions result in better functional improvement and higher satisfaction scores, underscoring the importance of personalized rehabilitation plans to meet the unique needs of each patient [30]. Complications and adverse events are major determinants of short-term outcomes following joint replacement surgeries. Common postoperative complications, such as infections, blood clots, and implant failures, can severely disrupt recovery. These complications often extend recovery times and reduce patient satisfaction. Moreover, the occurrence of adverse events not only delays healing but also negatively affects patients' perceptions of surgical success, resulting in lower PROM scores and overall dissatisfaction with the procedure [31]. Factors influencing short-term PROMs following total hip and knee replacement are shown in Table 1.

**Table 1:** Factors influencing short-term patient-reported outcome measures (PROMs) following total hip and knee replacement.

Category	Factor	Description	Impact on short-term PROMs
Patient-specific [32]	Age	Older age may be associated with slower recovery and higher complication rates.	Potentially lower physical function and quality of life scores.
	Gender	Women may report lower pain relief and physical function post-surgery compared to men.	Variations in pain perception and functional recovery.
	Preoperative health status	Comorbidities like diabetes or obesity can complicate recovery.	Reduced improvement in physical function and overall satisfaction.
	Mental health	Anxiety or depression can affect the perception of recovery and pain.	Lower scores in pain relief, quality of life, and patient satisfaction.
	Preoperative pain and function	Baseline pain levels and functional limitations before surgery.	Higher initial pain relief and functional gain post-surgery.
Surgical [33]	Type of implant	Different prosthesis designs and materials.	Variations in patient satisfaction and functional outcomes.
	Surgical approach	Techniques such as minimally invasive vs. traditional methods.	Differences in recovery time, pain levels, and mobility.
	Surgeon expertise	Experience and skill level of the surgeon.	Higher expertise is associated with better PROMs and fewer complications.
	Anesthesia type	General vs. regional anesthesia used during surgery.	Impact on immediate postoperative pain and recovery experience.
Rehabilitation [34]	Timing of rehabilitation	Early vs. delayed initiation of physical therapy.	Early rehabilitation often leads to better physical function and mobility.
	Intensity and duration	Duration and frequency of rehabilitation sessions.	More intensive programs can result in improved functional outcomes.
	Patient adherence	Compliance with prescribed rehabilitation protocols.	Higher adherence leads to better pain relief and physical function.
Complications [35]	Postoperative pain	Acute pain management strategies and effectiveness.	Poor pain management can lead to lower pain relief and satisfaction scores.
	Infection	Incidence of postoperative infections.	Infections can significantly impact mobility and overall satisfaction.
	Prosthesis-related issues	Dislocation, loosening, or wear of the implant.	Adversely affects physical function and patient satisfaction.
	Readmission and reoperation	Need for additional surgery or hospital readmission.	Negative impact on all domains of PROMs, especially satisfaction.

Current evidence: Evaluating short-term PROMs in THR and TKR

Recent studies and clinical trials have increasingly focused on evaluating short-term PROMs in THR and TKR. This literature review summarizes key findings, highlights variability across different patient populations, and compares outcomes between THR and TKR [26,36,37]. A study comparing anterior and posterior approaches in THR found that patients undergoing the direct anterior approach reported an improved recovery quality, underscoring the surgical technique's influence on short-term PROMs. Research analyzing PROMs following TKR has indicated that while overall satisfaction improves significantly post-surgery, notable differences in outcomes exist based on individual patient characteristics, such as age and pre-existing conditions [38]. Variability in short-term PROMs has been observed across different patient populations. Factors such as demographic differences, comorbidities, and psychological states can significantly affect recovery trajectories and satisfaction levels after surgery. A study focusing on negative outcomes after TKR highlighted the importance of understanding individual patient experiences to enhance future surgical outcomes and PROM validity across diverse populations [39]. THR and TKR show significant PROM improvements within the first six months post-surgery, with most patients reporting reduced pain and improved functional mobility. However, some studies suggest that TKR may have a steeper initial recovery curve compared to THR, possibly due to the complexities of knee mechanics versus hip mechanics [11]. Recovery trajectories differ between THR and TKR patients; THR patients often experience rapid improvements in mobility. TKR patients may face a more gradual recovery due to factors such as joint stiffness and challenges in pain management [11]. Enhanced recovery protocols have demonstrated benefits for both groups but may yield more pronounced effects in TKR patients, highlighting the necessity for tailored rehabilitation strategies based on joint type [40]. Current evidence suggests that while THR and TKR lead to significant short-term PROM improvements, variability exists across different patient groups. Understanding these differences is crucial for optimizing surgical techniques and postoperative care to enhance patient outcomes [40].

Challenges and limitations in assessing short-term PROMs

Assessing short-term PROMs presents several challenges and limitations that can significantly impact their effectiveness and reliability. One primary issue is the variability in PROM reporting, which can arise from individual patient characteristics such as age, gender, socioeconomic status, and psychological factors [41]. These elements often introduce biases in how patients perceive and report their outcomes. For instance, individuals from diverse backgrounds may interpret questions differently or possess varying levels of understanding regarding the importance of completing these measures. This variability can result in inconsistent data that complicates the evaluation of treatment effectiveness. Additionally, reporting bias may occur if patients feel pressured to provide socially desirable responses rather than accurately reflect their true health status, skewing results and diminishing the validity of the used PROMs [42]. Another significant limitation lies in the PROM tools themselves. Many instruments lack the sensitivity to detect changes across diverse patient populations. Most PROMs have been validated primarily in specific groups, which may not accurately reflect the experiences of broader or underrepresented populations [43]. For example, tools like PROMIS have demonstrated utility but may not adequately capture immediate changes in conditions or treatments for all demographic groups. This lack of sensitivity can hinder the ability to draw meaningful conclusions about treatment efficacy across different patient demographics [43]. Cultural and linguistic factors also play a crucial role in how patients interpret and complete PROMs. Instruments that have not been culturally adapted may lead to misunderstandings or misinterpretations of questions, resulting in inaccurate data collection [44]. For instance, translations that do not account for cultural nuances may fail to convey the intended meaning, negatively impacting patient responses. Furthermore, non-English speakers and individuals from different cultural backgrounds are often underrepresented in PROM data collection, raising concerns about the generalizability of findings and the potential for bias in understanding treatment outcomes across diverse populations [44]. Challenges and limitations in assessing short-term PROMs following total hip and knee replacement are shown in Table 2.

**Table 2:** Challenges and limitations in assessing short-term patient-reported outcome measures (PROMs) following total hip and knee replacement.

Category	Challenge/limitation	Description	Impact on assessment
Variability in reporting [45]	Patient self-reporting bias	Patients may overestimate or underestimate their symptoms and recovery due to personal perceptions or expectations.	Inconsistent or inaccurate PROM data affects overall conclusions.
	Recall bias	Patients may not accurately remember their preoperative condition or early postoperative experiences.	Distorted comparison between preoperative and postoperative scores.
	Cultural and linguistic differences	Differences in language and cultural interpretations can influence responses to PROM questionnaires.	Challenges in standardization and comparability of PROMs data.
Tool limitations [41]	Sensitivity to changes	Some PROMs may not be sensitive enough to detect subtle changes in specific patient subgroups or conditions.	Underestimation of recovery progress or treatment effects.
	Limited scope of domains	Certain PROMs may not capture all relevant aspects of recovery, such as mental health or social well-being.	Incomplete assessment of patient outcomes and quality of life.
	Ceiling and floor effects	PROMs may not detect improvements in patients who already have high scores (ceiling effect) or very low scores (floor effect).	Misleading interpretation of postoperative improvements.
Data collection issues [46]	Timing of assessments	Variability in the timing of PROMs collection (e.g., one month vs. three months post-surgery) can affect outcomes.	Difficulty in comparing results across studies with different timelines.
	Response rates and follow-up	Low response rates or loss to follow-up can result in incomplete data and potential bias.	Reduced reliability and generalizability of findings.
	Mode of administration	Differences in administration (e.g., paper vs. electronic) may affect patient responses and engagement.	Variation in data quality and patient compliance.
Interpretation challenges [47]	Minimal clinically important difference (MCID)	Difficulty in defining the smallest change in PROMs that patients perceive as beneficial.	Challenges in interpreting the clinical relevance of changes in PROMs scores.
	Heterogeneity of patient populations	Variability in patient characteristics (e.g., age, comorbidities) makes it difficult to generalize findings.	Complicates the interpretation and applicability of results to broader populations.
	Influence of expectations	Patient expectations can significantly influence reported outcomes and satisfaction.	Potential bias in PROMs scores based on preoperative expectations.

Future directions and recommendations

Future directions for improving PROMs in clinical practice emphasize standardization, personalization, and integrating longitudinal studies. A pressing need exists for standardized PROM tools and reporting guidelines to ensure consistency and reliability across clinical settings. Such standardization can enhance the comparability of data and improve the overall quality of patient care. By establishing uniform criteria for assessing outcomes, healthcare providers can more effectively evaluate the effectiveness of interventions and facilitate meaningful comparisons across studies [48]. In addition to standardization, integrating digital health technologies offers a significant opportunity to enhance PROMs. Mobile applications that enable real-time tracking of patient-reported outcomes can facilitate continuous monitoring and improve patient engagement. These digital tools provide timely feedback to healthcare providers and patients, allowing for more responsive care tailored to individual needs. By leveraging technology, clinicians can gain deeper insights into patient experiences and adjust treatment plans as necessary [49]. Personalization of PROM assessments is another critical area for future development. Tailoring PROMs to individual patient characteristics, such as demographics, comorbidities, and treatment goals, can enhance the relevance and accuracy of the measures. This customization ensures that assessments reflect the unique circumstances of each patient, ultimately improving satisfaction with care. By acknowledging that each patient's journey is distinct, healthcare providers can deliver more targeted interventions that align with specific patient needs [50]. Finally, there is a strong need for longitudinal studies that integrate short-term PROMs with long-term outcomes. Such studies are essential for comprehensively assessing treatment effectiveness over time. By linking immediate postoperative results with longer-term recovery trajectories, researchers can identify trends and patterns that inform better clinical decision-making [51]. This holistic approach not only enhances our understanding of surgical outcomes but also contributes to improved future patient care strategies. Together, these recommendations aim to enhance the utility of PROMs in clinical practice, ensuring they effectively capture patient experiences and outcomes while promoting personalized care strategies [51]. Future directions and recommendations for improving short-term PROMs following total hip and knee replacement are shown in Table 3.

**Table 3:** Future directions and recommendations for improving short-term patient-reported outcome measures (PROMs) following total hip and knee replacement.

Category	Recommendation	Description	Expected impact
Standardization [41]	Development of universal PROM tools	Create standardized PROM instruments that are validated across diverse populations and healthcare settings.	Enhanced comparability and consistency of data across studies and clinical settings.
	Standardized reporting guidelines	Establish uniform protocols for the timing and method of PROM data collection and reporting.	Improved data quality and reliability, facilitating meta-analyses and systematic reviews.
Digital integration [52]	Use of digital health technologies	Incorporate mobile apps, wearables, and online platforms for real-time tracking and collection of PROM data.	Increased patient engagement, real-time data collection, and improved adherence to follow-up.
	Remote monitoring and telemedicine	Utilize telemedicine for continuous monitoring of patients’ recovery and PROM tracking, especially in remote areas.	Enhanced patient access to care and continuous data collection post-surgery.
Personalization [48]	Customization of PROMs assessments	Develop patient-specific PROM tools considering individual health profiles, comorbidities, and personal goals.	More relevant and precise measurement of patient outcomes and satisfaction.
	Patient-centered outcome measures (PCOMs)	Incorporate measures that reflect patient preferences, expectations, and treatment goals.	Greater alignment of clinical care with patient needs and values.
Longitudinal studies [53]	Integration of short-term and long-term PROMs	Design studies that track patients’ outcomes from preoperative to long-term follow-up periods.	Comprehensive understanding of recovery trajectories and long-term benefits.
	Multi-center and cross-population studies	Conduct studies across different healthcare settings and patient demographics to validate findings.	Enhanced generalizability and applicability of PROMs data to diverse populations.
Clinical application [54]	Use of PROMs in shared decision-making	Integrate PROM data into clinical decision-making to guide treatment planning and patient counseling.	Improved patient satisfaction and outcomes through personalized care plans.
	PROMs as quality indicators	Use PROM data as key performance indicators for evaluating healthcare quality and surgical outcomes.	Enhanced focus on patient-centered care and quality improvement initiatives.
Research innovation [55]	Development of new PROM tools	Create new, more sensitive PROM tools that capture nuanced changes in patient recovery, particularly for diverse populations.	Better assessment of recovery progress and identification of specific patient needs.
	Validation of PROMs in diverse populations	Ensure existing and new PROM tools are validated for different cultural, linguistic, and demographic groups.	Increased accuracy and cultural relevance of PROMs data.

## Conclusions

In conclusion, evaluating short-term PROMs following THR and TKR is essential for understanding the immediate impact of these surgeries on patient satisfaction, functional recovery, and quality of life. PROMs provide invaluable insights into the patient’s perspective, complementing clinical and objective measures, and are crucial for assessing early postoperative success. The evidence suggests that short-term outcomes, such as pain relief, mobility, and physical function improvements, significantly influence overall recovery trajectories. However, patient-specific factors, surgical techniques, and postoperative care variations can impact these outcomes. By continuously refining and standardizing PROM tools, incorporating personalized assessments, and focusing on rehabilitation strategies, healthcare providers can enhance the quality of care and ensure that joint replacement surgeries meet the evolving needs of patients. Furthermore, integrating short-term PROMs with long-term follow-up data will provide a more comprehensive understanding of patient recovery and guide improvements in surgical practices and postoperative management.
